# C-C motif chemokine ligand 20 regulates neuroinflammation following spinal cord injury via Th17 cell recruitment

**DOI:** 10.1186/s12974-016-0630-7

**Published:** 2016-06-23

**Authors:** Jianzhong Hu, Zhiming Yang, Xiaoning Li, Hongbin Lu

**Affiliations:** Department of Spine Surgery, Xiangya Hospital, Central South University, 87 Xiangya Road, Changsha, 410000 People’s Republic of China; Department of Sports Medicine, Research Center of Sports Medicine, Xiangya Hospital, Central South University, 87 Xiangya Road, Changsha, 410000 People’s Republic of China

**Keywords:** Spinal cord injury, Neuroinflammation, CCL20, Th17, Chemokine, Flow cytometry

## Abstract

**Background:**

Spinal cord injury (SCI) is a severe traumatic injury that often leads to paralysis. The neuroinflammation following SCI plays an important role during the secondary injury phase. C-C motif chemokine ligand 20 (CCL20) works like a magnet to attract inflammatory cells and subsequently regulate inflammation. However, the role and mechanisms of CCL20 in neuroinflammation following traumatic injury are poorly understood.

**Methods:**

A modified Allen’s weight drop method was applied to induce a rat moderate contusion injury model. HE staining was used to assess spinal cord histopathology, and the water content test was used to estimate spinal cord edema. Motor function scores were quantified to evaluate locomotor ability, and leukocyte infiltration was observed by CD45 immunofluorescence and flow cytometry. Additionally, qRT-PCR and ELISA were used to determine inflammatory mediator gene expression. Th17 cell recruitment was identified by flow cytometry.

**Results:**

Compared with the injury control groups, histological analysis of the lesion area and tissue edema revealed reduced spinal cord edema and decreased lesion volume in the group administrated with CCL20 neutralizing antibody. Locomotor activity, as assessed by Basso, Beattie, and Bresnahan (BBB) score, showed that CCL20 blockade was beneficial for motor function recovery. Results also showed that leukocyte infiltration was reduced by neutralizing CCL20 at 7 days post-injury. More importantly, expression levels of IL-1β, IL-6, and TNF-α at 24 h after SCI demonstrated that a reduced inflammatory reaction in the CCL20 antibody group compared with the injury controls. Although CCL20 altered the expression of IL-1β, IL-6, and TNF-α, it had no effect on anti-inflammatory IL-10 expression at 24 h after damage. Notably, tissue flow cytometry confirmed that Th17 cell recruitment in the CCL20 antibody group was decreased compared with the control groups at 14 days post-injury. Additionally, IL-17A expression, which is mainly secreted by Th17 cell, suggested that CCL20 blockade also reduced IL-17A levels at 14 days after SCI.

**Conclusions:**

These results suggested that CCL20 aggravates neuroinflammation following SCI via regulation of Th17 cell recruitment and IL-17A level. Thus, CCL20-target therapy could be a promising clinical application for the treatment of SCI.

**Electronic supplementary material:**

The online version of this article (doi:10.1186/s12974-016-0630-7) contains supplementary material, which is available to authorized users.

## Background

Spinal cord injury (SCI) is often the result of a traffic accident, injury, or violence and has a devastating and complex impact on the central nervous system. The prevalence of SCI in the USA had reached 906 per million within the past decade and continued to show a significant growth trend [1]. By 2013, the National Spinal Cord Injury Statistical Center (NSCIS) reported that approximately 273,000 patients suffered from SCI in the USA, with 12,000 new cases in the USA annually [2]. SCI, which often leads to permanent disabilities such as paralysis and loss of movement or sensation, is characterized by primary injury from the physical trauma and secondary injury that may be amenable to therapy. The secondary injury following SCI is mainly induced by the activation of large numbers of cellular and molecular changes, including spinal cord neuroinflammatory reactions, vascular damage, neuronal and glial apoptosis, metabolic failure, and cellular excitotoxicity [3, 4]. Following disruption of the blood-spinal cord barrier, an influx of inflammatory cells and a strong expression of inflammatory mediators induce a reactive process of secondary cell death in tissue surrounding the original injury site [5, 6]. This inflammatory reaction continues for days or weeks and could ultimately lead to cavitation and formation of a glial scar, thereby exacerbating neurological dysfunction.

Numerous studies have demonstrated the mechanisms of neuroinflammation in SCI animal models and human samples [4, 7–10]. Alleviation of the early inflammatory response to SCI may limit the extent of tissue damage and, accordingly, the subsequent disability [11, 12]. Despite extensive experimental data suggesting that neuroinflammation is a pathogenic component of SCI, the inflammatory reaction also appears to be vital for tissue repair [13]. Recent studies have illustrated that the neuroinflammation following SCI exhibits neuroprotective effects on neurons and glia, and this effect was beneficial for tissue repair [14, 15]. It remains a challenge for researchers to determine how to control crosstalk between the nervous system and the immune system to ease neurodegeneration while promoting axonal regeneration. The exploration of therapeutic targets and suitable time points during neuroinflammation has become a hot topic in the SCI field.

A growing body of evidence in animal models has confirmed that T lymphocytes play an important role in spinal cord injury. Gonzalez et al. showed that reduced T lymphocyte recruitment through the use of a chemoattractant CXCL10 neutralizing antibody significantly enhances spinal cord tissue preservation and functional outcomes [16]. CD4^+^ T-helper (Th) cells are mediators involved in the response to traumatic injury in the central nervous system (CNS). Fee and his colleagues found that activated CD4^+^ Th cells exacerbate acute traumatic brain injury in immunodeficient mice [17]. As a subgroup of Th cells producing interleukin-17 (IL)-17, Th17 cells play a role in inflammation and tissue injury. Fossiez demonstrated that IL-17 mediates the release of IL-6 and IL-8, and these cytokines reflect the response to inflammation and recruitment of neutrophils into tissues [18]. Increasing evidence shows that Th17 cells are involved in the inflammatory damage of nephritis, dry eye disease, and acute viral myocarditis [19–21]. However, the role that Th17 cells play in the pathogenesis of SCI remains unknown.

Chemokines work like magnets to direct movements of immune cells by binding to specific chemokine receptors expressed at the cell surface. Subsequently, the attracted immune cells arrive at their intended destination [22, 23]. C-C motif chemokine ligand 20 (CCL20), also known as MIP-3α and LARC, is a small cytokine that is mainly released by neuron, astrocyte, and microglia in CNS. It is noteworthy that CCL20 can attract not merely lymphocyte but also monocyte and dendritic cell. As the unique receptor of CCL20, C-C chemokine receptor 6 (CCR6) is preferentially expressed by Th17 cells [24, 25]. Additionally, recent studies showed that CCL20/CCR6 interactions direct Th17 cell migration, which ultimately leads to progression of immune-mediated diseases [19–21, 26]. Several studies also suggested that CCL20 plays a pivotal role in inflammatory cascades in brain injury and cerebral ischemia [27–29].

In the present study, we hypothesized that CCL20 directs Th17 cell migration to the injury site and alters neuroinflammation within the SCI. We used in vivo neutralization of CCL20 to demonstrate the functional relevance of CCL20 in mediating the inflammatory response after SCI. Finally, Th17 cell recruitment was evaluated to determine the mechanism of CCL20 in the regulation of neuroinflammation following SCI.

## Methods

### Animals

Adult, male, Sprague-Dawley (SD) rats weighing 180 to 220 g were provided by the Experimental Animal Center of Central South University (Changsha, China) and were housed in individual cages in a climate-controlled room with free access to water and food.

### Animal model of contusion SCI

Anesthesia was induced with 4 % isoflurane and maintained with 2 % isoflurane in 98 % O_2_. Throughout the procedure, the depth of sedation was monitored by an absent response to a toe pinch. A laminectomy was performed at the thoracic vertebra level 10 (T10) after shaving and cleaning in a warm environment (Additional file 1: Figure S1). A moderate contusion injury was induced using a modified Allen’s weight drop apparatus (8 g weight at a vertical height of 40 mm, 8 g × 40 mm) on the spinal cord, as previously described [30]. Sham-operated animals were only subjected to laminectomy. After surgery, the muscles were sutured in layers and the skin incision was closed with 3-0 silk threads. Penicillin G (40,000 U, i. m.) was administrated daily for 3 days to prevent infection. Abdominal massage was conducted twice daily to help in the recovery of bladder function until full voluntary or autonomic voiding was obtained. Rats that died for any reasons were excluded from the experiment, and a new one was added to the study. The mortality rate of this study was approximately 2 %.

### Experimental groups and interventions

The rats were randomly assigned to four groups: sham-operated rats (sham group), anti-CCL20 monoclonal neutralizing antibody-treated SCI rats (CCL20 mAb group), mouse immunoglobulin (Ig) G1 antibody-treated SCI rats (isotype control group), and SCI rats that received no treatment (SCI group). As previously described, the anti-rat CCL20 antibody (MAB540; R&D Systems Inc., Minneapolis, MN, USA) and mouse IgG1 isotype control antibody (MAB002; R&D Systems) were dissolved in phosphate-buffered saline (PBS) and diluted to 100 μg/ml [29]. CCL20 mAb group or isotype control group rats were administrated an intraperitoneal injection of CCL20 antibody (100 μg/kg body weight) or isotype control antibody (100 μg/kg body weight) immediately after surgery, respectively.

### Behavioral assessment

Three rats from each group were subjected to locomotor activity evaluation at 1, 3, 7, 14, 21, and 28 days post-injury using the Basso, Beattie, and Bresnahan (BBB) score method. Two independent and well-trained testers observed movement of each rat for 4 min and scored motor functions according to BBB scales [31]. The final score for each animal was obtained by averaging values from both investigators. Rats with perineal infections, limb wounds, or tail and foot grazing were eliminated from the test.

### Tissue preparation

For the quantitative real-time polymerase chain reaction (qRT-PCR), enzyme-linked immunosorbent assay (ELISA), and flow cytometry, rats were sacrificed by transcardiac perfusion with cold PBS to eliminate RNA and protein expressed by blood cells. The spinal cord was immediately dissected on ice. Thereafter, 10-mm-long spinal cord segments containing the injury epicenter were removed as quickly as possible. The samples, except for the flow cytometry samples, were then flash-frozen and stored in liquid nitrogen for subsequent RNA and protein extraction. Lymphocytes were isolated from the fresh spinal cord for flow cytometry. For immunohistochemistry (IHC), immunofluorescence (IF), and hematoxylin and eosin (H&E) staining, rats were sacrificed by transcardiac perfusion with PBS followed by 4 % paraformaldehyde. The 10-mm-long injured spinal cords were then carefully isolated and post-fixed in 4 % paraformaldehyde for 24 h at 4 °C. Specimens were then immersed in 15 and 30 % sucrose solution for 12 h, respectively. After fixation and dehydration, the spinal cords were paraffin-embedded and processed into 6-μm transverse sections. For the water content test, animals were sacrificed without transcardiac perfusion, and 15-mm spinal tissues were obtained at the edge of the injury site. Spinal cord weight was subsequently measured using an electronic analytical balance. Three rats from each group were subjected to mouse IgG measurements from 0 h to 28 days post-injury by ELISA. Rat blood sample was drawn from caudal vein and then centrifuged at 3000 rpm for the collection of serum.

### qRT-PCR analysis

The three spinal cords from each group at 1 and 14 days after damage were used to measure mRNA levels. Total RNA was obtained from 10-mm-long spinal cord samples and extracted using TRIzol reagent (Invitrogen, Carlsbad, CA, USA) according to manufacture instructions. Total RNA from each specimen was reverse-transcribed to cDNA employing the PrimeScript RT reagent kit with gDNA Eraser (Takara, Tokyo, Japan), and qRT-PCR was conducted using the SYBR Premix Ex Taq (Takara). GAPDH expression was used as an internal control, and expression levels of rat CCL20, IL-1β, IL-6, TNF-α, IL-10, and IL-17A were expressed as fold increases or decreases compared with the control. Gene expression analyses were quantified using the 2^**−ΔΔ**Ct^ method, in which mRNA levels from the sham group were used as controls. Primer sequences for qPCR were detailed in Additional file 2: Table S1.

### ELISA analysis

The three spinal cords of each group at 1 and 14 days post-injury were used to detect cytokine protein levels by ELISA according to manufacturer’s instructions (Cusabio Biotech Co, Wuhan, China). Blood samples of each group from 0 h to 28 days post-SCI were also used to measure mouse IgG levels dynamically by ELISA according to manufacture instructions (Cusabio Biotech Co.). All assays were performed in duplicates using recommended buffers, diluents, and substrates. Immunoreactivity was determined by a microplate reader at 450 nm (Multiskan FC Microplate Photometer, Thermo Fisher Scientific Inc., Rockford, IL, USA). The tissue cytokine concentrations were expressed as pg protein/ml while mouse IgG levels were expressed as ng protein/ml.

### Spinal cord water content measurement

The three spinal cords of each group were used to measure spinal cord water content using the wet-dry weight method as previously reported to evaluate spinal cord edema [32]. Briefly, rats were sacrificed at 72 h post-injury, the time point at which peak spinal cord edema develops after SCI according to a previous report [33]. The 15-mm lesioned spinal tissue was immediately dissected and weighed before and after drying in an electrothermostatic blast oven at 95 °C for 48 h. The percentage of water content was calculated using the formula: [(wet weight − dry weight) / wet weight] × 100 %. Spinal cord water content was measured by investigators blinded to the experimental groups.

### Lesion volume quantification by H&E staining

After the final locomotor function test and BBB scoring, which occurred 28 days after surgery, the spinal cord transverse sections (6 μm thickness) from the width of the spinal lesion site were stained with H&E. The sections were then traced using the Image-Pro Plus software program (Media Cybernetics, Rockvillie, MD, USA), and the lesion area and spared tissue area were outlined and quantified. Spared tissue was reported as the remaining areas where normal spinal cord structure was preserved. Conversely, the lesioned tissue was defined as injury areas where normal anatomical spinal cord structure was not preserved. The section with the greatest percentage of lesioned tissue area was assigned as the injury epicenter. Transverse sections, with an interval of 400 or 1600 μm rostral and caudal to the lesion epicenter, were analyzed for percentage tissue injury and sparing.

### Immunofluorescence staining

The three spinal cords from each group at 7 days after injury were used to observe leukocyte infiltration. The sections were dewaxed and rehydrated, and antigens were retrieved by heating in 10 mM citrate buffer (pH 6.0) for 10 min at 98 °C. The sections were then incubated with 10 % normal goat serum for 30 min at room temperature to block nonspecific staining. Immediately after, the sections were incubated overnight at 4 °C with rabbit anti-CD45 primary antibody (1:100, ab10558, Abcam, USA). After three PBS wash steps, the sections were then incubated for 1 h with a goat anti-rabbit fluorescein secondary antibody (Alexa Fluor 488, 1:400, Jackson ImmunoResearch, West Grove, PA). Finally, the nuclei were counterstained with DAPI and observed as soon as possible. Images were analyzed by Image-Pro Plus software to quantify positive cells and total cells in five random fields of each section. The semi-quantitative expression of CD45 was assessed as percentage of positive cells per field = (positive cells / total cells) × 100 %.

### Immunohistochemistry staining

Transverse sections 3 mm rostral to the injury epicenter were obtained from three rats of each group 1 and 14 days post-injury. In brief, paraffin-embedded sections (6 μm thickness) were deparaffinized with xylene and hydrated through a graded alcohol step. Antigen retrieval was performed by microwave irradiation in 10 mM citrate buffer (pH 6.0) twice for 5 min at 800 W prior to cooling for 30 min. Subsequently, 3 % H_2_0_2_ was used to block endogenous peroxidase activity for 15 min at room temperature, and then sections were rinsed three times for 5 min each with 0.01 M PBS. Afterwards, sections were blocked with 10 % normal goat serum for 1 h to reduce nonspecific staining and then incubated overnight at 4 °C with primary antibodies: rabbit anti-macrophage inflammatory protein 3 alpha polyclonal antibody (1:200, ab9829, Abcam) and rabbit anti-IL-17A polyclonal antibody (1:100, orb48920, Biorbyt, UK). After PBS rinse steps, the sections were incubated for 30 min at room temperature with goat anti-rabbit secondary antibody (1:800, ab6721, Abcam). Subsequently, immunoreactivity was visualized by staining with diaminobenzidine (DAB) for 3 min under a light microscope. Finally, sections were counterstained with hematoxylin for 1 min and then analyzed using Image-Pro Plus software. The brown staining indicated positive expression of the corresponding protein. Percentage of positive cells was calculated identical to the immunofluorescence protocol.

### Flow cytometry

The nine rats from each group were subjected to quantification of CD45-positive cell infiltration and Th17 cell recruitment (three rats performed one test). Immediately after removing the 10-mm-long spinal cord segment containing the injury epicenter, the samples were placed in a RPMI 1640 (Gibco) for single cell suspension preparation as previously described [34]. Cell suspensions were prepared by mechanical dissociation and were forced through a 70-μm cell strainer (Falcon, USA). Afterwards, myelin was removed following centrifugation on 37 % isotonic Percoll (GE Healthcare Bio-Sciences AB, Uppsala, Sweden). Isolated spinal cord lymphocytes were suspended in 1 ml cell staining buffer and stained by trypan blue to count live cell. The live cell frequencies of all samples were greater than 95 %. Lymphocytes for CD45 flow cytometry were stained with cell surface markers: anti-rat CD45 (0.125 μg, PE, eBioscience, USA) or rat IgG1 K isotype control (0.125 μg, PE, eBioscience). In addition, lymphocytes for Th17 cell flow cytometry were then stimulated with a cell stimulation cocktail (1:500, eBioscience, USA) in an incubator (37 °C, 5 % CO_2_) for 6 h. The cells were washed and stained with cell surface markers: anti-rat CD3 (0.25 μg, APC, eBioscience) and anti-rat CD8a (0.25 μg, FITC, eBioscience). After that, the Fix/Perm buffer (eBioscience) was used for fixation and permeabilization, followed by intracellular staining with anti-rat IL-17A (0.125 μg, PE, eBioscience) or rat IgG2a K isotype control (0.125 μg, PE, eBioscience). Finally, the lymphocytes were analyzed with a Becton Dickinson FACSCalibur system using FlowJo software (version 7.6; TreeStar, Inc., Ashland, OR, USA). Initially, CD3^+^ cells (T lymphocytes) were gated by vs. CD3 APC as the T-gate protocol. Additionally, owing to endocytosis of CD4 molecules on the surface of T cells after IL-17 stimulation, the CD3^+^ cells were analyzed for CD8 FITC expression to counter-target the CD3^+^4^+^ (Th) cells. This specific cell population was then evaluated for intracellular IL-17A PE expression vs. CD3^+^8^−^. Thus, Th17 cells were presented as CD3^+^CD8^−^IL-17A^+^ cells.

### Statistical analysis

All data are based on at least three independent experiments. The data are expressed as mean ± SD, except for data from the behavioral assessment of BBB scores, which is presented as mean ± SEM. Statistical analyses were performed using SPSS 17.0 (SPSS Inc., Chicago, IL, USA). Data from the BBB scores were analyzed using repeated measures analysis of variance (ANOVA). For other data, statistical comparisons were analyzed using *t* test or one-way ANOVA followed by a Student-Newman-Keuls test. A *P* value less than 0.05 was considered to be statistically significant.

## Results

### Altered spatiotemporal level of CCL20 in the spinal cord after SCI

**Fig. 1:**
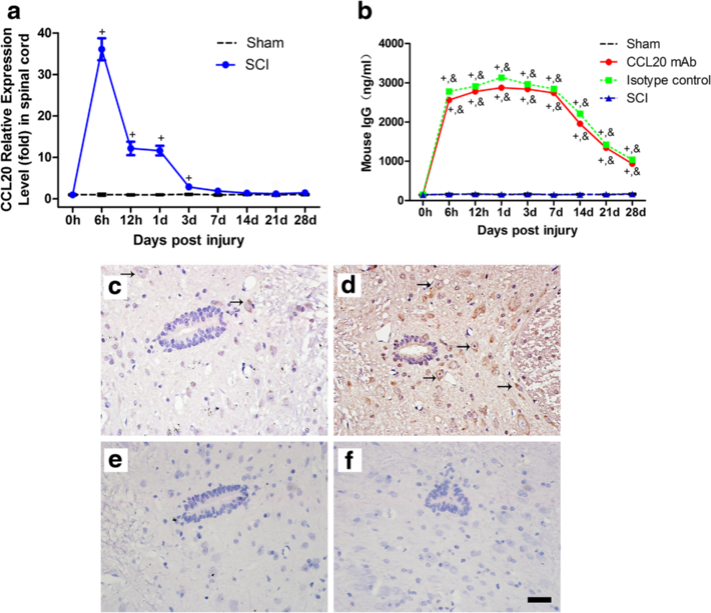
Altered spatiotemporal level of CCL20 in the spinal cord after SCI. **a** The temporal profile (from 0 h to 28 days post-injury) of CCL20 mRNA expression in the spinal cord, as determined by qRT-PCR, shows that SCI leads to increased CCL20 mRNA level in the spinal cord, especially during the early period of SCI. **b** Mouse IgG levels of rat serum (from 0 h to 28 days post-injury), as determined by ELISA, are significantly increased in CCL20 mAb group and isotype control group from 6 h to 28 days post-SCI. CCL20 immunostaining at 1 day post-injury in the sham group **(c)**, SCI group **(d)**, negative control of the sham group **(e)**, and negative control of the SCI group **(f)** indicates that CCL20 is mainly localized in the cytoplasm of gray matter neurons and glial cells. The brown staining represents positive CCL20 expression. *Black arrow* indicates the CCL20 positive neuron and glial cell. Scale bar = 100 μm. ^+^ *P* < 0.05, compared with the sham group; ^*^ *P* < 0.05, compared with CCL20 mAb group; &*P* < 0.05, compared with SCI group

We measured CCL20 expression levels in the spinal cord at different time points from 0 h to 28 days post-injury using qRT-PCR (Fig. 1a). CCL20 expression increased in the SCI group and reached a peak level at 6 h after damage, and then gradually declined to baseline at 7 days post-injury. As shown in Fig. 1b, the mouse IgG level of rat serum, as determined by ELISA from 0 h to 28 days post-injury, significantly increased in the CCL20 mAb group and isotype control group from 6 h to 28 days post-SCI when compared with sham group and SCI group. These data suggested that SCI leads to increased CCL20 expression in the spinal cord, especially during the early period of SCI. Additionally, CCL20 monoclonal neutralizing antibody may persist for the entire period of the observation even at the late time point (28 days) where we evaluate neurological outcome and histopathological outcome of SCI.

Immunohistochemistry was applied to investigate spatial expression of CCL20 in the spinal cord at 1 day post-injury. Results showed that CCL20 was mainly localized in the cytoplasm of gray matter neurons and glial cells, which was in accordance with previous observations (Fig. 1c–f) [27]. By showing temporal and spatial CCL20 level in the spinal cord with or without injury, these results demonstrated that CCL20 is a promising target worthy of study.

### CCL20 blockade improves neurological outcomes after SCI

**Fig. 2:**
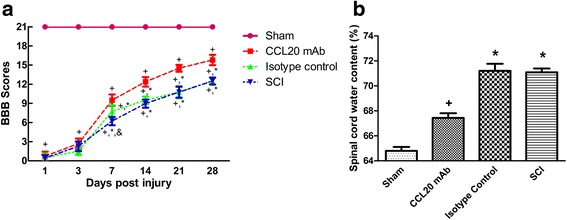
CCL20 blockade improves neurological outcome after SCI. **a** Analysis of locomotor BBB scores after SCI (from 1 to 28 days post-injury). A significant improvement in hindlimb motor function is observed in the CCL20 mAb group compared with the injury control groups from 7 days post-injury. **b** The water content data shows that CCL20 blockade significantly alleviates spinal cord edema at 3 days post-SCI. ^+^ *P* < 0.05, compared with the sham group; ^*^ *P* < 0.05, compared with the CCL20 mAb group; &*P* < 0.05, compared with the isotype control group

The impacts of CCL20 on neurological outcomes of SCI rats were assessed by the behavioral evaluation from BBB scores and the severity of spinal cord edema from water content test, which are closely related to clinical disabilities. Following establishment of the contusion SCI rat model, all animals were paralyzed in both hindlimbs. We found spontaneous functional recovery after SCI in all injury groups. As shown in Fig. 2a, there was no difference in BBB scores between injury groups at 1 and 3 days post-injury, indicating that rats in different groups had relatively comparable injuries. During the observation period, as illustrated by increased BBB scores, hindlimb locomotor activity gradually improved. Compared with the SCI group or isotype control group, motor function recovery significantly increased in the CCL20 mAb group from 7 days post-injury, demonstrating that CCL20 blockade was beneficial for movement recovery in SCI rats.

Spinal cord water content tests were performed to evaluate edema severity at 72 h post-injury, the time period when peak spinal cord edema develops after SCI. As shown in Fig. 2b, water content of the SCI group and isotype control group significantly increased, whereas edema severity was significantly reduced in the CCL20 mAb group (71.09 ± 0.31 and 71.20 ± 0.57 %, respectively, vs. 67.44 ± 0.37 %). These results demonstrated that neutralizing CCL20 resulted in ameliorated spinal cord edema.

Taken together, these data showed that CCL20 blockade improves motor function recovery and alleviates spinal cord edema. CCL20 itself may exacerbate hindlimb motor function deterioration and spinal cord edema in SCI rats.

### Neutralizing CCL20 improves histopathological outcomes in the injured spinal cord

**Fig. 3:**
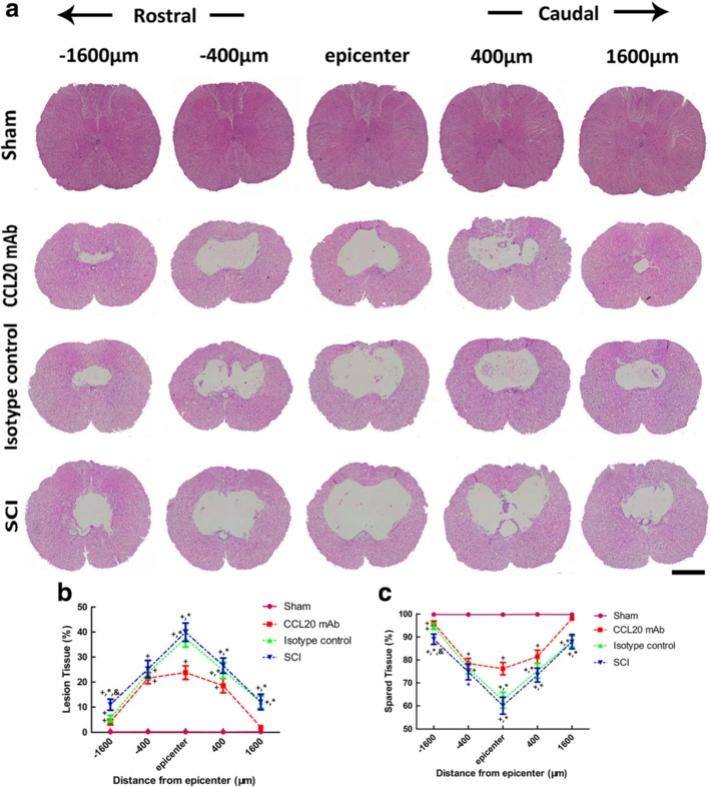
Neutralizing CCL20 improves histopathological outcome in the injured spinal cord. **a** H&E staining of the spinal cord sections at 28 days post-SCI shows varying sizes of the cystic cavity, inflammatory cell infiltration, and formation of a glial scar around the cavity. Scale bar = 1 mm. **b, c** Quantitative data of lesion tissue and spared tissue shows a significantly decreased lesion volume and increased spared tissue area, respectively, in CCL20 mAb-treated rats at the injury epicenter and both rostral and caudal directions, respectively. ^+^ *P* < 0.05, compared with the sham group; ^*^ *P* < 0.05, compared with the CCL20 mAb group; &*P* < 0.05, compared with the isotype control group

H&E staining was applied to evaluate spinal cord lesion size and tissue sparing following behavioral assessment at 28 days post-injury. Varying sizes of the cystic cavity, inflammatory cell infiltration, and the formation of a glial scar around the cavity was observed in the injury groups (Fig. 3a). We also determined the lesion area and spared tissue area size of the injured spinal cord using Image-Pro Plus software. Compared to the SCI group or isotype control group, there was significantly decreased lesion volume or increased spared tissue area in the CCL20 mAb-treated rats at the injury epicenter and in regions extending away from the epicenter in both rostral and caudal directions (Fig. 3b, c). It is worth noting that the results were consistent with BBB scores at 28 days post-injury, illustrating that histopathological outcome correlated with neurological outcome.

### CCL20 neutraliztion reduces leukocyte infiltration

**Fig. 4:**
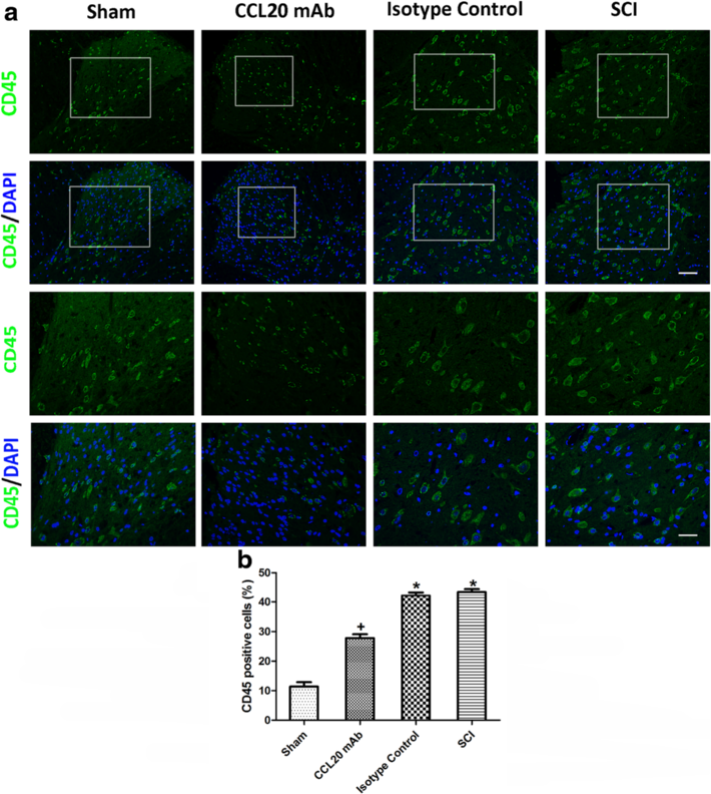
CCL20 neutralization reduces leukocyte infiltration. **a** Immunostaining of the spinal cord sections at 7 days post-SCI shows various amounts of CD45-positive cells in the different groups. CD45-positive cells are stained *green*, while the nuclei are stained *blue*. The immunostained areas in the *white box* are magnified below. Scale bars are 50 and 100 μm, respectively. **b** The percentage of CD45-positive cells indicates that leukocyte infiltration, as well as neuroinflammation during secondary injury, was significantly alleviated by neutralizing CCL20. ^+^ *P* < 0.05, compared with the sham group; ^*^ *P* < 0.05, compared with the CCL20 mAb group

**Fig. 5:**
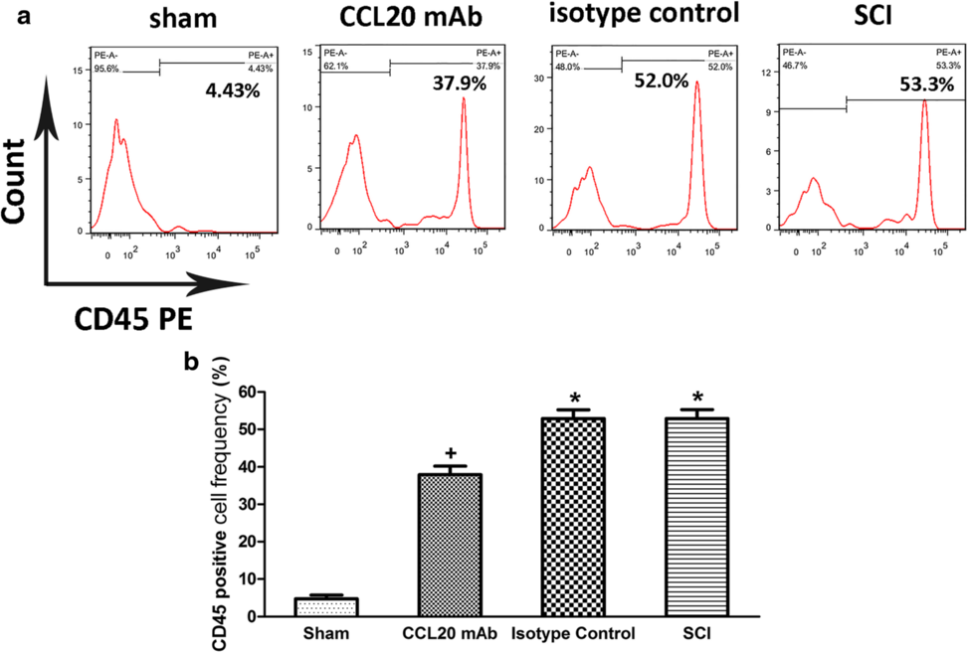
Neutralizing CCL20 reduces leukocyte infiltration. **a** Flow cytometry of the spinal cord at 7 days post-SCI shows the CD45^+^ cell of different groups. **b** The frequency of CD45^+^ cell indicates that leukocyte infiltration was significantly alleviated by CCL20 neutralization. ^+^ *P* < 0.05, compared with the sham group; ^*^ *P* < 0.05, compared with the CCL20 mAb group

As a marker of extravasated leukocyte, CD45, also known as leukocyte common antigen, was used to evaluate leukocyte infiltration and inflammatory response at 7 days post-injury [35]. Very little CD45 expression was observed in the sham-operated rats. Sections from the SCI group and isotype control group showed abundant CD45 immunostaining, whereas the CD45-positive cell infiltration was attenuated by CCL20 mAb (Fig. 4a). As shown in Fig. 4b, the percentage of CD45-positive cells was 11.39 ± 1.49 % in the sham group, 27.83 ± 1.29 % in the CCL20 mAb group, 42.27 ± 1.06 % in the isotype control group, and 43.47 ± 1.02 % in the SCI group, which demonstrated that CCL20 mAb administration significantly reduced CD45^+^ cell accumulation. On the other hand, we also detected CD45^+^ cell frequency by flow cytometry. As shown in Fig. 5, compared with the SCI group or isotype control group, anti-CCL20 antibody administration significantly decreased CD45^+^ cell frequency at 7 days post-injury. These results indicated that leukocyte infiltration, as well as neuroinflammation during secondary injury, was significantly alleviated by CCL20 blockade.

### Neutralizing CCL20 attenuates local inflammatory response and decreases expression of pro-inflammatory cytokines

**Fig. 6:**
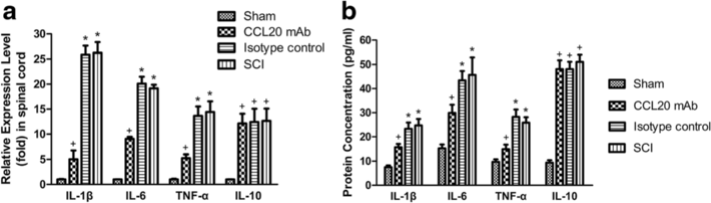
Neutralizing CCL20 decreases the expression of pro-inflammatory cytokines. **a** The qRT-PCR data at 24 h post-injury shows that treatment with neutralizing anti-CCL20 antibody significantly decreased mRNA expression of IL-1β, IL-6, and TNF-α. However, CCL20 mAb had no effect on the expression of the anti-inflammatory IL-10. **b** Similarly, ELISA data at 24 h post-injury illustrates that CCL20 blockade significantly reduced protein expression of IL-1β, IL-6, and TNF-α but had no influence on the expression of IL-10. ^+^ *P* < 0.05, compared with the sham group; ^*^ *P* < 0.05, compared with the CCL20 mAb group

The local inflammation during secondary injury of SCI was evaluated by expression of IL-1β, IL-6, TNF-α, and IL-10 at 24 h post-injury. Expression of IL-1β, IL-6, TNF-α, and IL-10 mRNA was quantified using qRT-PCR. Compared with the SCI group or isotype control group, treatment with neutralizing anti-CCL20 antibody significantly decreased IL-1β, IL-6, and TNF-α expression at 24 h post-injury (Fig. 6a). Although, CCL20 altered the expression of pro-inflammatory cytokines, it had no influence on expression of the anti-inflammatory IL-10 at 24 h after damage. Expression of IL-1β, IL-6, TNF-α, and IL-10 protein was measured using ELISA. Similarly, CCL20 neutralization significantly decreased IL-1β, IL-6, and TNF-α expression compared with the injury control groups at 24 h after SCI but had no significant effect on IL-10 expression (Fig. 6b).

Overall, these results indicated that the neuroinflammatory reaction of the CCL20 blockade group was milder than the injury controls at 24 h post-injury. Although CCL20 altered the expression of IL-1β, IL-6, and TNF-α, it had no influence on the expression of the anti-inflammatory IL-10 at 24 h post-injury.

### Th17 cells are recruited by CCL20 after SCI

**Fig. 7:**
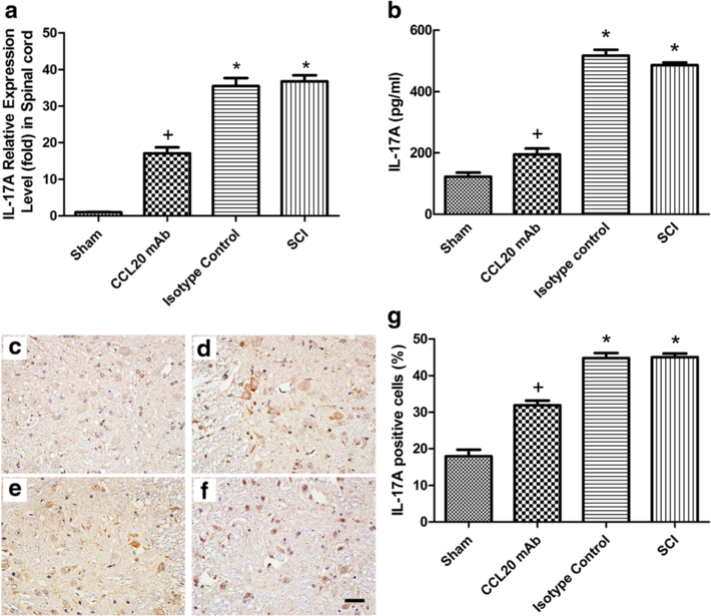
CCL20 blockade attenuates the expression of IL-17A. **a, b** The qRT-PCR and ELISA data at 14 days post-injury show that mRNA and protein expression of IL-17A, respectively, significantly decreased as a result of treatment with CCL20 mAb. IL-17A immunostaining at 14 days post-SCI in the sham group **(c)**, CCL20 mAb group **(d)**, isotype control group **(e)**, and SCI group **(f)** indicates that IL-17A was mainly localized in the cytoplasm of lymphocytes and neurons. The *brown staining* represents the positive expression of IL-17A. Scale bar = 100 μm. **g** The percentage of IL-17A-positive cells indicates that CCL20 neutralizing antibody significantly reduced the IL-17A secretion. ^+^ *P* < 0.05, compared with the sham group; ^*^ *P* < 0.05, compared with the CCL20 mAb group

To determine the amount of Th17 cell accumulation in the injured spinal cord, we measured IL-17A mRNA and protein expression, which potentially represents the amount of Th17 cells at 14 days post-injury. We analyzed IL-17A expression at 14 days post-injury, because mRNA expression of IL-17A culminated on day 14, as shown by qRT-PCR (Additional file 3: Figure S2). As shown in Fig. 7a, SCI induced a dramatic increase in IL-17A expression, whereas this elevation was attenuated by CCL20 neutralization. Similarly, IL-17A protein expression significantly increased in the isotype control group and SCI group following contusion injury, as measured by ELISA. However, treatment with CCL20 neutralizing antibody significantly reduced IL-17A protein expression (Fig. 7b).

Results showed that IL-17A was mainly localized in the cytoplasm of the spinal cord lymphocytes and neurons (Fig. 7c–f). As shown in Fig. 7g, the percentage of IL-17A-positive cells was significantly reduced by CCL20 blockade compared with the isotype control group and SCI group (31.88 ± 1.31 vs. 44.77 ± 1.36 and 45.02 ± 0.99 %, respectively). These results indicated that IL-17A expression was significantly decreased by neutralizing CCL20. It could be postulated that CCL20 might increase IL-17A expression by recruitment of Th17 cells. Thus, lymphocytes were separated from the spinal cords of rats at 14 days post-injury and analyzed by flow cytometry to illustrate the direct relationship between CCL20 and Th17 cells.

**Fig. 8:**
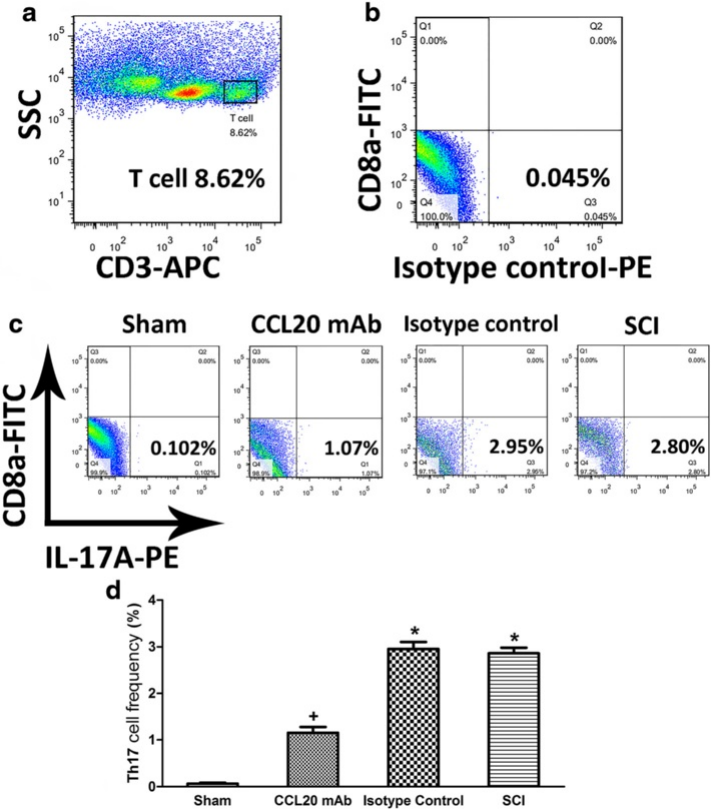
Th17 cells are recruited by CCL20 post-SCI. **a** Th17 cells from the spinal cord at 14 days post-injury were initially targeted by CD3^+^ T cells from isolated lymphocytes. **b** The isotype control from the IL-17A-PE staining. **c** CD8^−^ IL-17A^+^ cells represent Th17 cells. **d** The frequency of Th17 cell was significantly reduced by application of CCL20 mAb, indicating that CCL20 blockade directly decreases Th17 cell recruitment in the spinal cord. ^+^ *P* < 0.05, compared with the sham group; ^*^ *P* < 0.05, compared with the CCL20 mAb group

We initially targeted CD3^+^ T cells from isolated lymphocytes (Fig. 8a). Subsequently, the isotype control-PE was used for the isotype control in the IL-17A-PE staining (Fig. 8b). In Fig. 8c, the CD8^−^ IL-17A^+^ cells represented the Th17 cells. As shown in Fig. 8d, the frequency of Th17 cell significantly increased in the isotype control group and SCI group compared with the sham group (2.96 ± 0.15 and 2.86 ± 0.12 %, respectively, vs. 0.06 ± 0.02 %). However, the frequency of Th17 cell significantly decreased to 1.15 ± 0.12 % in the CCL20 mAb group, indicating that CCL20 blockade directly reduced recruitment of Th17 cells in the spinal cord.

Taken together, these data demonstrated that neutralizing CCL20 reduced Th17 cell infiltration and subsequent secretion of IL-17A. CCL20 itself may induce recruitment of Th17 cells and subsequently expression of IL-17A.

## Discussion

SCI induces a series of robust immune responses via secretion of cytokines and chemokines and a combined infiltration of peripheral leukocytes into the injured area. Cytokines, chemokines, and leukocytes were the key factors that observed in the present study to evaluate inflammatory responses following SCI. Neuroinflammation during secondary injury may result in a further exacerbation of neurological function outcome, because of the formation of scar tissue and necrosis or apoptosis of neurons and oligodendrocytes. Unlike other tissues, this may give rise to permanent functional loss in the CNS, because of the extremely limited ability to repair damaged axons and replenish lost neurons [4]. Of note, Louveau and colleagues recently discovered functional lymphatic vessels lining the dural sinuses, and these vessels carried both fluid and immune cells from the cerebrospinal fluid [36]. The spinal cord may also has lymphatic vessels that transport immune cells, which further supports the theory that immune responses play a role in neuroinflammation following SCI. However, numerous studies over the past decade have revealed that post-traumatic inflammation can play a dual role. Neuroinflammation, except for the deleterious effects, also exhibits a neuroprotective effect on neurons and glia and is beneficial to the initiation of tissue repair. Therefore, the time course after SCI becomes a vital factor to manipulate pro- and anti-inflammatory responses to minimize tissue damage and to promote tissue repair, respectively.

Initially, the inflammatory response post-SCI is dominated by the recruitment of neutrophils that pass through the broken blood-spinal cord barrier, this recruitment peaks at 1 day post-injury. Subsequently, blood-derived macrophages and activated CNS resident microglia dominate the neuroinflammation from day 2 onwards. Shortly afterwards, lymphocytes, although in small numbers, start to infiltrate into the injured spinal cord. The major detrimental effect of neuroinflammation after SCI is likely to occur within the first 2 weeks [11, 37]. Thus, drug-targeted therapies to regulate the inflammatory response following SCI would be appropriate only for a limited period after injury. Therefore, the present study investigated the possibilities of CCL20-tageted intervention for the treatment of SCI only during the acute period. Spinal cord decompression surgery and drug therapies have limited benefits for SCI recovery. The aim of the surgery is to create a comfortable environment for self-healing of the injured spinal cord, but the self-repair is very slow and uncontrollable. Methylprednisolone was broadly accepted to treat SCI a decade ago. However, the safety and efficacy of methylprednisolone has been more recently criticized. Therefore, it is necessary to determine neuroinflammatory therapeutic targets and corresponding time points for alleviation of detrimental bystander effects and facilitation of beneficial aspects.

CCL20 caught our attention, because its mRNA expression increased almost 37-fold at 6 h after SCI (Fig. 1a). In the present study, we first investigated the impact of CCL20 on neurological outcome after SCI. We found that CCL20 blockade in the spinal cord is beneficial for improving neurological function in SCI animals, as indicated by ameliorated spinal cord edema, improved long-term movement function, and decreased lesion volume (Figs. 2 and 3). These data are similar to studies focused on brain injury [28, 29]. Because CCL20 expression increased in the spinal cord from 6 h to 7 days post-injury in the SCI group (Fig. 1a), we hypothesized that CCL20 neutralization is a protective response following SCI and that CCL20 could serve as a potential therapeutic target to improve recovery following SCI.

CCL20, as a chemokine, regulates inflammatory responses by recruiting Th17 cells, which has been previously shown in animal models of IgA nephropathy, dry eye disease, and acute viral myocarditis [19–21]. Therefore, we analyzed the role of CCL20 in spinal cord neuroinflammation after SCI. Results suggested that CCL20 blockade resulted in reduced leukocyte infiltration and expression of pro-inflammatory cytokines (Figs. 4, 5, and 6), further indicating that neutralizing CCL20 alleviated neuroinflammation following SCI. We also observed that CCL20 had no effect on the expression of the anti-inflammatory cytokine IL-10, indicating that CCL20 may regulate neuroinflammation by directly manipulating IL-1β, IL-6, and TNF-α expression, but not IL-10. These results have not been previously shown in other studies of spinal cord injury or brain injury. Furthermore, we studied the mechanism of CCL20 during the inflammatory response after SCI by measuring Th17 cells and expression of IL-17A. We found that CCL20 blockade results in decreased recruitment of Th17 cells and subsequent IL-17A expression in SCI rats (Figs. 7 and 8). Meanwhile, results from mouse IgG of rat serum reflected that CCL20 mAb administrated in vivo may persist for the entire period of the observation even at the late time point (28 days) where we evaluate neurological outcome and histopathological outcome of SCI (Fig. 1b). In addition, persistent neutralizing CCL20 after SCI may gradually reduce leukocyte infiltration, pro-inflammatory cytokine expression, and consequently neuroinflammation. The alleviation of neuroinflammation which plays an important role in secondary injury mechanism may finally influence the neurological and histopathological outcomes in the later stage of SCI.

**Fig. 9:**
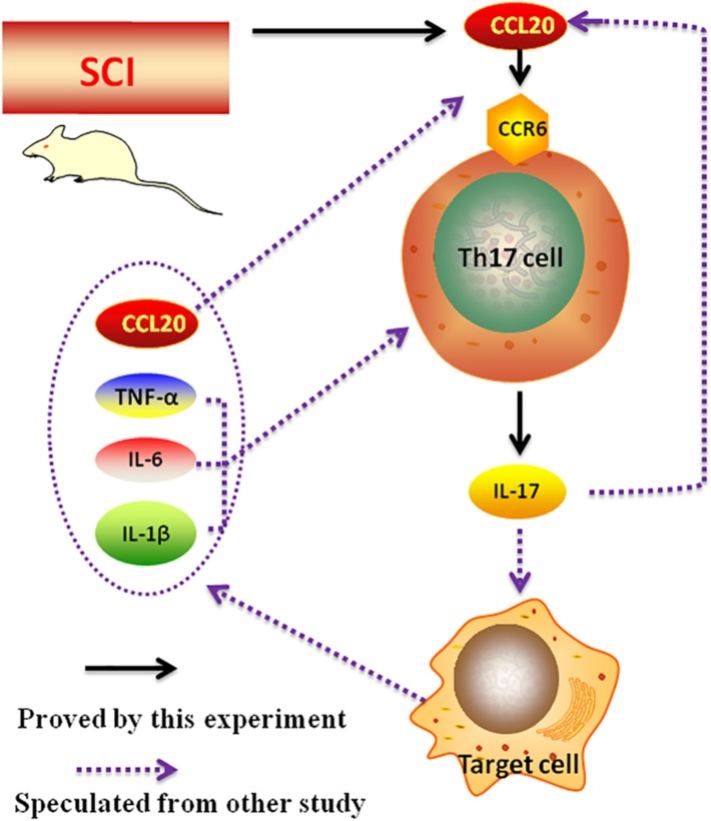
The postulated inflammatory positive feedback in neuroinflammation following SCI. IL-17 interacts with the corresponding receptors on the surfaces of certain target cells and induces the production of IL-6, IL-1β, TNF-α, and CCL20. Subsequently, IL-6, IL-1β, and TNF-α further stimulate IL-17 expression and differentiation of Th17 cells. The *black solid line* represents what we have proven in the present study. The *purple dotted line* represents what we have speculated from other studies

Based on numerous studies, Fossiez demonstrated that IL-17 mediates the release of IL-6 and IL-8, and these cytokines reflect inflammation and recruitment of neutrophils into tissues [18]. Miossec and Kolls concluded that IL-17 also induces production of CCL20, IL-1β, and TNF-α, which implicates IL-17 in the exacerbation of inflammatory responses [38]. Additionally, IL-1β and TNF-α is thought to prompt Th17 cell development by enhancing the effect of IL-17 on mRNA stability, leading to increased levels of protein expression [39]. Bettelli indicated that IL-6 is a key factor in initial differentiation of Th17 cells [40]. Therefore, together with results from Morishima [41], we postulated that IL-17 interacts with corresponding receptors on the surfaces of certain target cells and induce production of IL-6, IL-1β, TNF-α, and CCL20. Subsequently, IL-6, IL-1β, and TNF-α stimulate the expression of IL-17 and differentiation of Th17 cells, suggesting an inflammatory positive feedback between Th17 cells and the induced pro-inflammatory cytokines (Fig. 9). Thus, CCL20-based therapy is an effective intervention to disrupt this inflammatory positive feedback from the initial source.

Results from the present study demonstrated that CCL20 regulates neuroinflammation following SCI via Th17 cells recruitment and subsequent IL-17A expression. However, further studies are needed to determine the remaining mechanisms involved in the postulated inflammatory positive feedback. Although important discoveries were revealed by the present study, there were also limitations. It should be noted that this study examined only the impact of CCL20 on Th17 cells and IL-17 expression but did not address interactions between IL-17 and CCL20. We also used CCL20 neutralizing antibody to block CCL20 rather than stimulation of CCL20 bioactivity. These data may only partially reflect the role and mechanism of CCL20 in neuroinflammation following SCI. We could not ascertain from this data whether the observed alleviated neuroinflammation was only due to reduced recruitment of Th17 cells or whether other mechanisms were also at play. Moreover, it is insufficient to investigate the role of CCL20 only by administration of CCL20 mAb immediately after SCI.

Taken together, results from the present study proved the hypothesis that CCL20 directs Th17 cell migration to the injury site and alters neuroinflammation after SCI. This study expands on our previous understanding about the functions and mechanisms of CCL20 in neuroinflammation following CNS injury, suggesting that it could be a potential therapeutic target for post-SCI interventions. This research also opens a new avenue of therapeutic strategies for SCI by manipulating immune responses.

## Conclusions

SCI induces significant upregulation of CCL20, and neutralizing CCL20 in vivo improves functional recovery, attenuates tissue damage, and alleviates neuroinflammation in a rat SCI model. Moreover, these results suggest that CCL20 aggravates neuroinflammation following SCI via regulation of Th17 cell recruitment and IL-17A expression. Thus, CCL20-target therapy could be a promising clinical application for the treatment of SCI.

## Abbreviations

CCL20, C-C motif chemokine ligand 20; SCI, spinal cord injury; Th, T-helper; CNS, central nervous system; IL, interleukin; TNF, tumor necrosis factor; MIP, macrophage inflammatory protein; LARC, liver and activation-regulated chemokine; CCR6, C-C chemokine receptor 6; T10, thoracic vertebra Level 10; mAb, monoclonal antibody; Ig, immunoglobulin; PBS, phosphate-buffered saline; BBB score, Basso, Beattie, and Bresnahan score; qRT-PCR, quantitative real-time polymerase chain reaction; ELISA, enzyme-linked immunosorbent assay; IHC, immunohistochemistry; IF, immunofluorescence; H&E, hematoxylin and eosin; DAPI, 4′,6-diamidino-2-phenylindole; ANOVA, analysis of variance.

## Additional files

Additional file 1: Figure S1. Animal model of contusion SCI. (A) The localization of T10 by X-ray. (B) Skin preparation. (C) Skin incision. (D) The exposure of T10. (E) The exposure of the spinal cord. (F) Contusion injury. (G) Observation after SCI. (H) Suture the incision. (DOCX 2344 kb) Additional file 2: Table S1. Primer sequences for qPCR. Primers were designed and provided by Takara Bio Inc. (Tokyo, Japan). (DOCX 12 kb) Additional file 3: Figure S2. The temporal profile (from 0 h to 28 days post-injury) of IL-17A mRNA expression in the spinal cord. IL-17A, as determined by qRT-PCR, shows that SCI leads to increased IL-17A mRNA level in the spinal cord, especially at 14 days post-SCI. ^+^ *P* < 0.05, compared with the sham group. (DOCX 168 kb)
