# Supplementary material for: C-C motif chemokine ligand 20 regulates neuroinflammation following spinal cord injury via Th17 cell recruitment
Source: J Neuroinflammation. 2016 Jun 23;13:162. doi: 10.1186/s12974-016-0630-7 (PMC4918039; doi:10.1186/s12974-016-0630-7)
Supplement: Additional file 1: Figure S1. — Animal model of contusion SCI. (A) The localization of T10 by X-ray. (B) Skin preparation. (C) Skin incision. (D) The exposure of T10. (E) The exposure of the spinal cord. (F) Contusion injury. (G) Observation after SCI. (H) Suture the incision. (DOCX 2344 kb) [file 12974_2016_630_MOESM1_ESM.docx]

**Additional file 1: Figure S1**


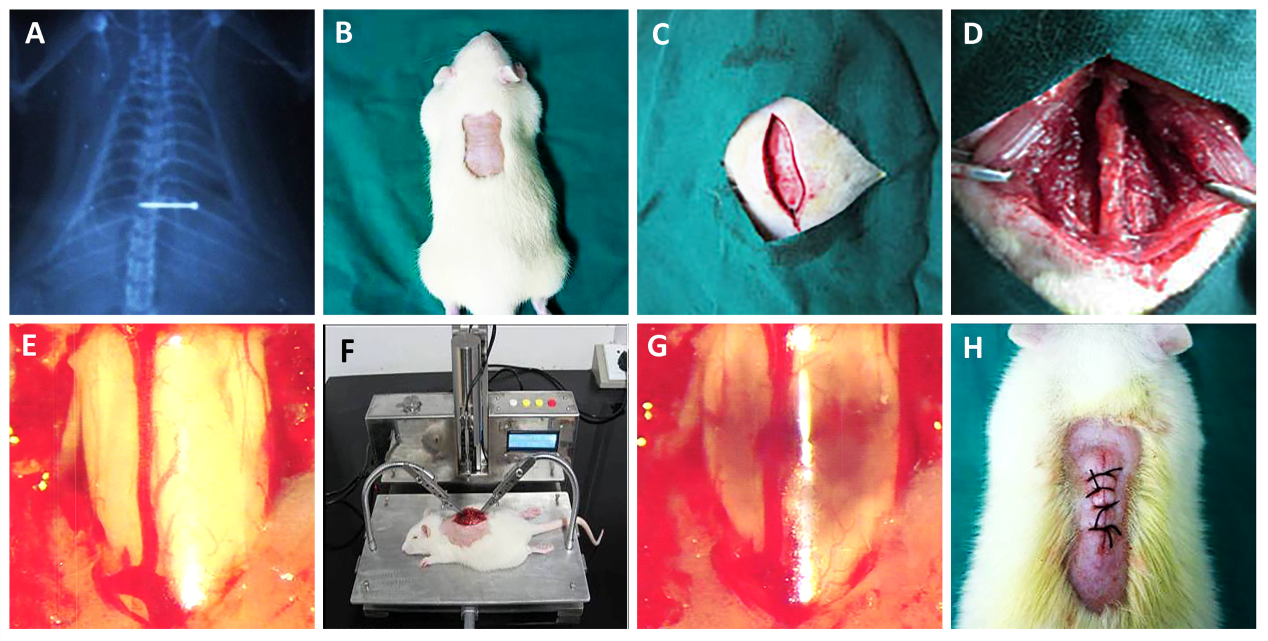


**Additional file 1: Figure S1. Animal** **model of contusion SCI. (A)** The localization of T10 by X-ray. **(B)** Skin preparation. **(C)** Skin incision. **(D)** The exposure of T10. **(E)** The exposure of spinal cord. **(F)** Contusion injury. **(G)** Observation after SCI. **(H)** Suture the incision.
