# Supplementary material for: C-C motif chemokine ligand 20 regulates neuroinflammation following spinal cord injury via Th17 cell recruitment
Source: J Neuroinflammation. 2016 Jun 23;13:162. doi: 10.1186/s12974-016-0630-7 (PMC4918039; doi:10.1186/s12974-016-0630-7)
Supplement: Additional file 2: Table S1. — Primer sequences for qPCR. Primers were designed and provided by Takara Bio Inc. (Tokyo, Japan). (DOCX 12 kb) [file 12974_2016_630_MOESM2_ESM.docx]

**Additional file 2: Table S1**

| **Marker** | **Forward primer** | **Reverse primer** |
| --- | --- | --- |
| *CCL20*  *IL-1β*  *IL-6*  *TNF-α*  *NF-κB*  *IL-17a*  *IL-10*  *GAPDH* | 5’-CACTGAGCAGATCAATTCCTGGAG-3’  5’-CCCTGAACTCAACTGTGAAATAGCA-3’  5’-ATTGTATGAACAGCGATGATGCAC-3’  5’-TCAGTTCCATGGCCCAGAC-3’  5’-TCTTCGACTACGCGGTTACGG-3’  5’-CTGATCAGGACGAGCGACCA-3’  5’-CAGACCCACATGCTCCGAGA-3’  5’-GGCACAGTCAAGGCTGAGAATG-3’ | 5’-TGTACGTGAGGCAGCAGTCAAAG-3’  5’-CCCAAGTCAAGGGCTTGGAA-3’  5’-CCAGGTAGAAACGGAACTCCAGA-3’  5’-GTTGTCTTTGAGATCCATGCCATT-3’  5’-CTCACGAGCTGAGCATGAAGG-3’  5’-ACTGTAGCCTCCAGGTTCAGTAGCA-3’  5’-CAAGGCTTGGCAACCCAAGTA-3’  5’-ATGGTGGTGAAGACGCCAGTA-3’ |

**Additional file 2: Table S1.** Primer sequences for qPCR. Primers were designed and provided by Takara Bio INC (Tokyo, Japan).
