# Supplementary material for: C-C motif chemokine ligand 20 regulates neuroinflammation following spinal cord injury via Th17 cell recruitment
Source: J Neuroinflammation. 2016 Jun 23;13:162. doi: 10.1186/s12974-016-0630-7 (PMC4918039; doi:10.1186/s12974-016-0630-7)
Supplement: Additional file 3: Figure S2. — The temporal profile (from 0 h to 28 days post-injury) of IL-17A mRNA expression in the spinal cord. IL-17A, as determined by qRT-PCR, shows that SCI leads to increased IL-17A mRNA level in the spinal cord, especially at 14 days post-SCI. + P < 0.05, compared with the sham group. (DOCX 168 kb) [file 12974_2016_630_MOESM3_ESM.docx]

**Additional file 3: Figure S2**


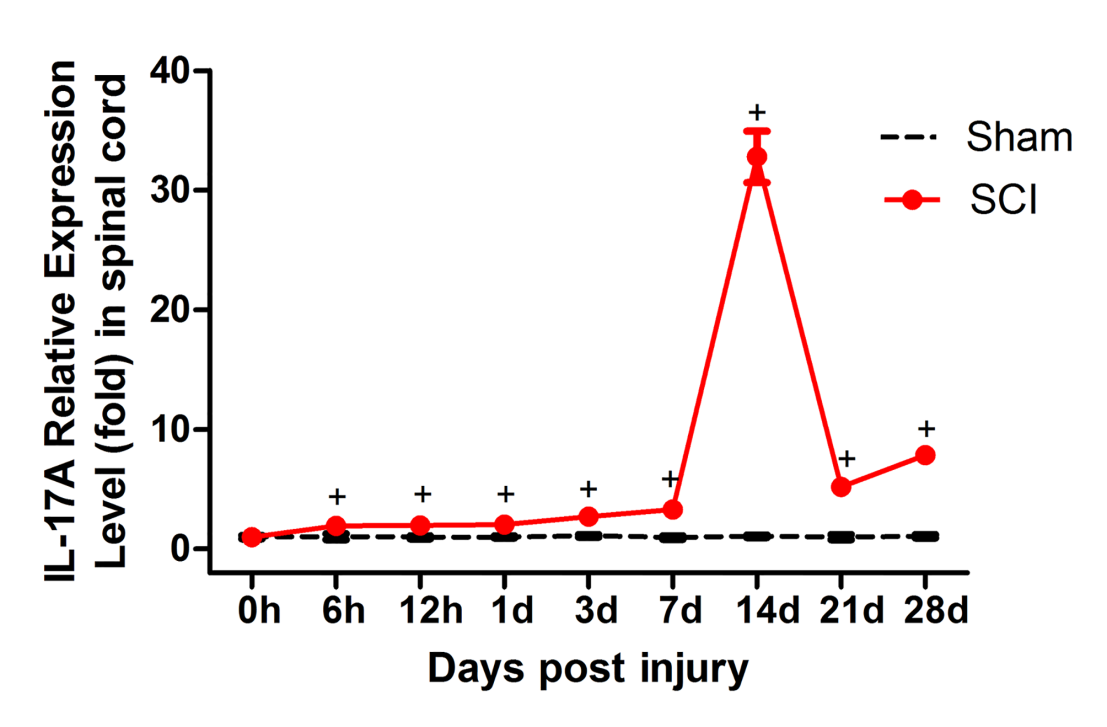


**Additional file 3: Figure S2. The temporal profile (from 0 h to 28 d post-injury) of IL-17A mRNA expression in the spinal cord.** IL-17A, as determined by qRT-PCR, shows that SCI leads to increased IL-17A mRNA level in the spinal cord, especially at 14 d post-SCI. ^+^*P* < 0.05, compared with sham group.
